# EZH2-mediated *Puma* gene repression regulates non-small cell lung cancer cell proliferation and cisplatin-induced apoptosis

**DOI:** 10.18632/oncotarget.10841

**Published:** 2016-07-26

**Authors:** Haidan Liu, Wei Li, Xinfang Yu, Feng Gao, Zhi Duan, Xiaolong Ma, Shiming Tan, Yunchang Yuan, Lijun Liu, Jian Wang, Xinmin Zhou, Yifeng Yang

**Affiliations:** ^1^ Clinical Center for Gene Diagnosis and Therapy, The Second Xiangya Hospital of Central South University, Changsha, Hunan, China; ^2^ Department of Cardiovascular Surgery, The Second Xiangya Hospital of Central South University, Changsha, Hunan, China; ^3^ Department of Thoracic Surgery, The Second Xiangya Hospital of Central South University, Changsha, Hunan, China; ^4^ Department of Radiology, The Third Xiangya Hospital of Central South University, Changsha, Hunan, China; ^5^ Hunan Cancer Hospital and The Affiliated Cancer Hospital of Xiangya School of Medicine, Central South University, Changsha, Hunan, China; ^6^ Department of Ultrasonography, The Third Xiangya Hospital of Central South University, Changsha, Hunan, China; ^7^ Department of Hemopathology, The Third Xiangya Hospital of Central South University, Changsha, Hunan, China; ^8^ Albert Einstein College of Medicine, Bronx, NY, USA

**Keywords:** polycomb repressive complex 2, EZH2, PUMA, non-small cell lung cancer, cisplatin

## Abstract

Polycomb group (PcG) proteins are highly conserved epigenetic effectors that maintain the silenced state of genes. EZH2 is the catalytic core and one of the most important components of the polycomb repressive complex 2 (PRC2). In non-small cell lung cancer (NSCLC) cells and primary lung tumors, we found that PRC2 components, including EZH2, are overexpressed. High levels of EZH2 protein were associated with worse overall survival rate in NSCLC patients. RNA interference mediated attenuation of EZH2 expression blunted the malignant phenotype in this setting, exerting inhibitory effects on cell proliferation, anchorage-independent growth, and tumor development in a xenograft mouse model. Unexpectedly, we discovered that, in the suppression of EZH2, p53 upregulated modulator of apoptosis (PUMA) expression was concomitantly induced. This is achieved through EZH2 directly binds to the *Puma* promoter thus epigenetic repression of PUMA expression. Furthermore, cisplatin-induced apoptosis of EZH2-knocking down NSCLC cells was elevated as a consequence of increased PUMA expression. Our work reveals a novel epigenetic regulatory mechanism controlling PUMA expression and suggests that EZH2 offers a candidate molecular target for NSCLC therapy and EZH2-regulated PUMA induction would synergistically increase the sensitivity to platinum agents in non-small cell lung cancers.

## INTRODUCTION

Lung cancer is the leading cause of cancer-related mortality among both men and women worldwide [1]. Non-small cell lung cancer (NSCLC), which includes adenocarcinoma, squamous-cell carcinoma, and large-cell lung cancer, comprises approximately 85% of all lung cancers [2]. The current treatment options for patients with advanced NSCLC are limited to chemotherapy and radiotherapy. Despite the development of new therapeutic agents and improved patient care, the 5-year survival rate of NSCLC is still dismal at around 15% [2, 3]. Novel therapeutic interventions are critical for improvement of the survival and prognosis of NSCLC patients.

The conserved epigenetic effector polycomb group (PcG) proteins, which are involved in control of gene expression, play critical roles in biologic processes, such as cellular development and tumorgenesis [4]. PcG proteins comprise two well-known polycomb repressive complexes 1 and 2 (PRC1 and PRC2), which are best characterized in the repression of transcription. The core components of PRC2 include enhancer of zeste homolog 2 (EZH2), suppressor of zeste 12 (SUZ12) and embryonic ectoderm development (EED) [5]. EZH2, which possesses histone methyltransferase (HMTase) activity, is required for PRC2-mediated gene repression via inducing of histone H3 methylation of lysine 27. EED physically associates with EZH2 and histone H3 and thus functions as a scaffold protein. SUZ12 is required for the nucleosome recognition, activity and stability of the PRC2 complex [6, 7]. EZH2 is a key component of the PCR2 complex and is frequently overexpressed in a wide variety of human malignancies such as breast [8], prostate [9], gastric [10] and lung cancers. Moreover, several studies have evaluated the role of EZH2 overexpression in NSCLC and demonstrated that high EZH2 expression is correlated with the early pathogenesis, tumor progression, poor prognosis and poor overall survival for patients with NSCLC [11–14]. EZH2 is therefore considered to be an oncogene. In addition, SUZ12 is also reportedly overexpressed in several human tumors including those of the colon, breast and liver [15]. The PRC2 maintains and establishes its target gene silencing during carcinogenesis [16, 17]. Thus, the PRC2 complex, especially its EZH2 subunit, appears to be an attractive target for therapeutic intervention. However, the PRC2 target genes specifically repressed in cancer cells and the mechanism whereby the PRC2 complex promotes tumor progression has not yet been fully determined.

PUMA is a member of the BH3-only Bcl-2 family and a potent inducer of apoptosis [18]. It binds to all five antiapoptotic Bcl-2 family members, such as BCL2, BCL-XL and MCL-1, which relieves their inhibition of BAK and BAX, results in the permeabilization of mitochondrial membrane, and subsequently caspase cascade activation [19]. PUMA induction is regulated by not only p53-dependent but also p53-independent mechanisms [20, 21]. For example, p53-dependent regulation of PUMA is dysfunctional in most cancer cells due to p53 abnormalities, causing survival of tumor cells and therapeutic resistance [21]. On the other hand, several transcription factors, including p65 [22], p73 [23] and FoxO3a [21, 24], have been implicated in p53-independent PUMA induction. However, epigenetic regulation of *Puma* gene expression in NSCLC cells remains unclear.

In the present study, we found that EZH2 plays an important role in lung tumorigenesis. Knockdown of EZH2 dramatically inhibited proliferation of NCSLC cells both *in vitro* and *in vivo*. In addition, PUMA expression was concomitantly induced upon the suppression of EZH2, which is achieved through EZH2 directly binds to the *Puma* promoter and thus epigenetic repression of PUMA expression in NSCLC cells. Furthermore, cisplatin-induced apoptosis of EZH2-knocking down NSCLC cells was elevated as a consequence of increased PUMA expression. Our results suggested that EZH2 offers a candidate molecular target for NSCLC therapy and EZH2-modulated PUMA induction would synergistically increase the sensitivity to platinum agents in NSCLCs.

## RESULTS

### PRC2 components are overexpressed in human non-small cell lung cancer

**Figure 1:**
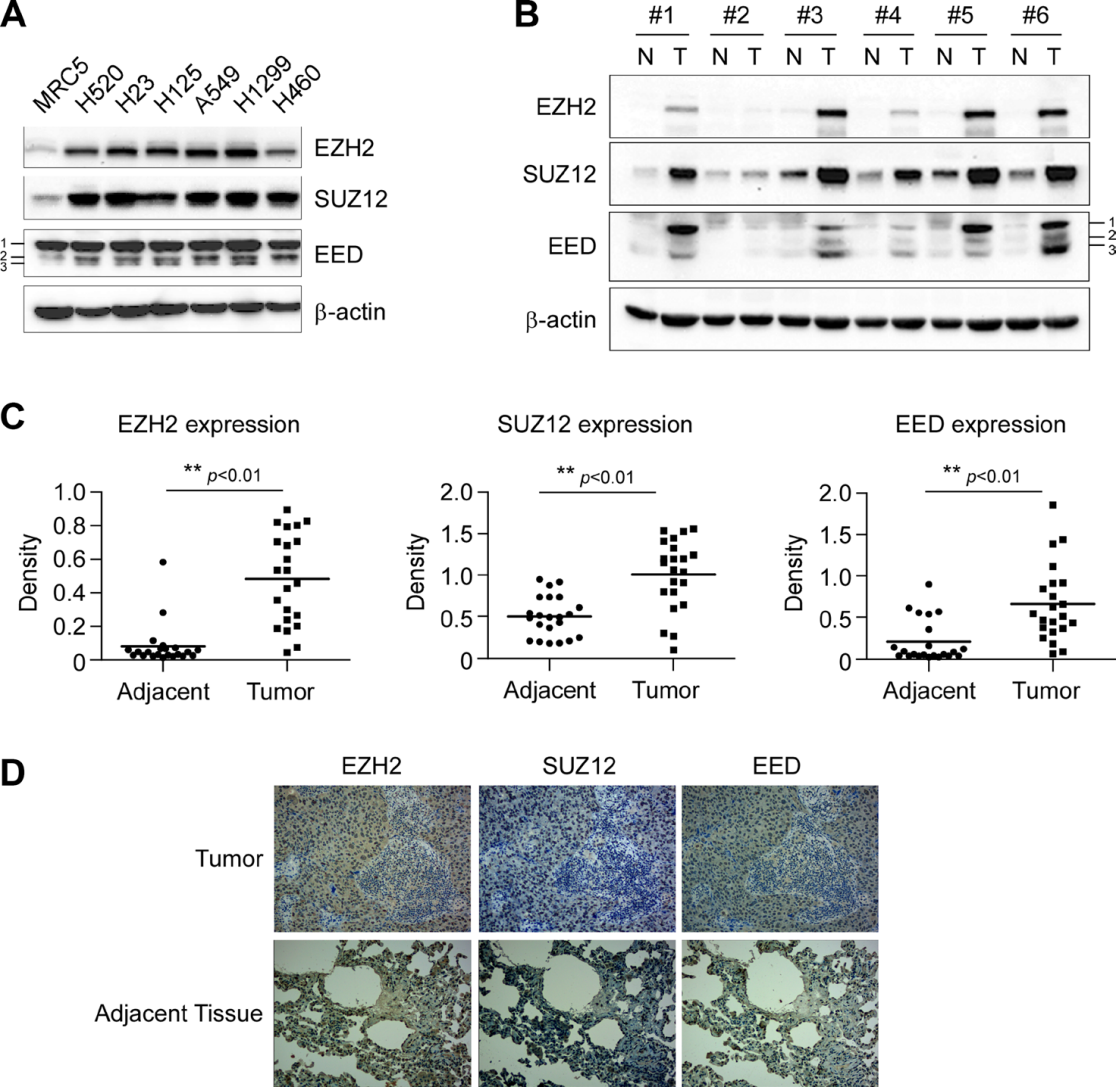
Aberrant overexpression of PRC2 proteins EZH2, SUZ12 and EED in human non-small cell lung cancer (**A**) PRC2 components EZH2, SUZ12 and EED are highly expressed in NSCLC cells. Western blot analysis was performed to examine EZH2, SUZ12 and EED expression in several NSCLC cell lines and normal MRC5 lung cells. EED isoforms are numbered. β-actin was used as a loading control. (**B**, **C** and **D)**. EZH2, SUZ12 and EED are highly expressed in human NSCLC tissues. EZH2, SUZ12 and EED protein levels in six representative NSCLC cases were assessed by Western blot analysis. β-actin was used as a loading control. N, adjacent normal tissues; T, tumor (B). Western blotting determined EZH2, SUZ12 and EED protein levels in malignant and the corresponding normal adjacent tissues of 22 NSCLC patients. The intensity was evaluated using Image J (NIH) computer software. **p <0.01, significant difference between groups as indicated (C). Representative figures of immunohistochemical staining for EZH2, SUZ12 or EED were performed on NSCLC tissues and the corresponding normal adjacent samples (D).

To investigate whether the high expression of PRC2 components is linked to tumorgenesis of NSCLC, the expression levels of EZH2, EED and SUZ12 were tested by western blotting in cultures of human fetal lung fibroblast cells MRC5 and six human NSCLC cell lines. When compared to MRC5 cells, EZH2, EED and SUZ12 were expressed at higher levels in all NSCLC cell lines examined (Figure 1A). We next sought to determine the protein levels of EZH2, EED and SUZ12 in human NSCLC specimens and matched adjacent normal tissue via western blotting. In matched normal adjacent samples, EZH2, EED and SUZ12 were not detectable or at a very low level (Figure 1B, 1C and 1D). On the contrary, EZH2, EED and SUZ12 were substantially overexpressed in tumor samples (*n* = 22, *p* < 0.01) (Figure 1B, 1C and 1D). These results indicated that PRC2 components EZH2, SUZ12 and EED might be critical molecules in NSCLC development.

### Knockdown of EZH2 inhibits the proliferation of human NSCLC cells *in vitro* and *in vivo*

**Figure 2:**
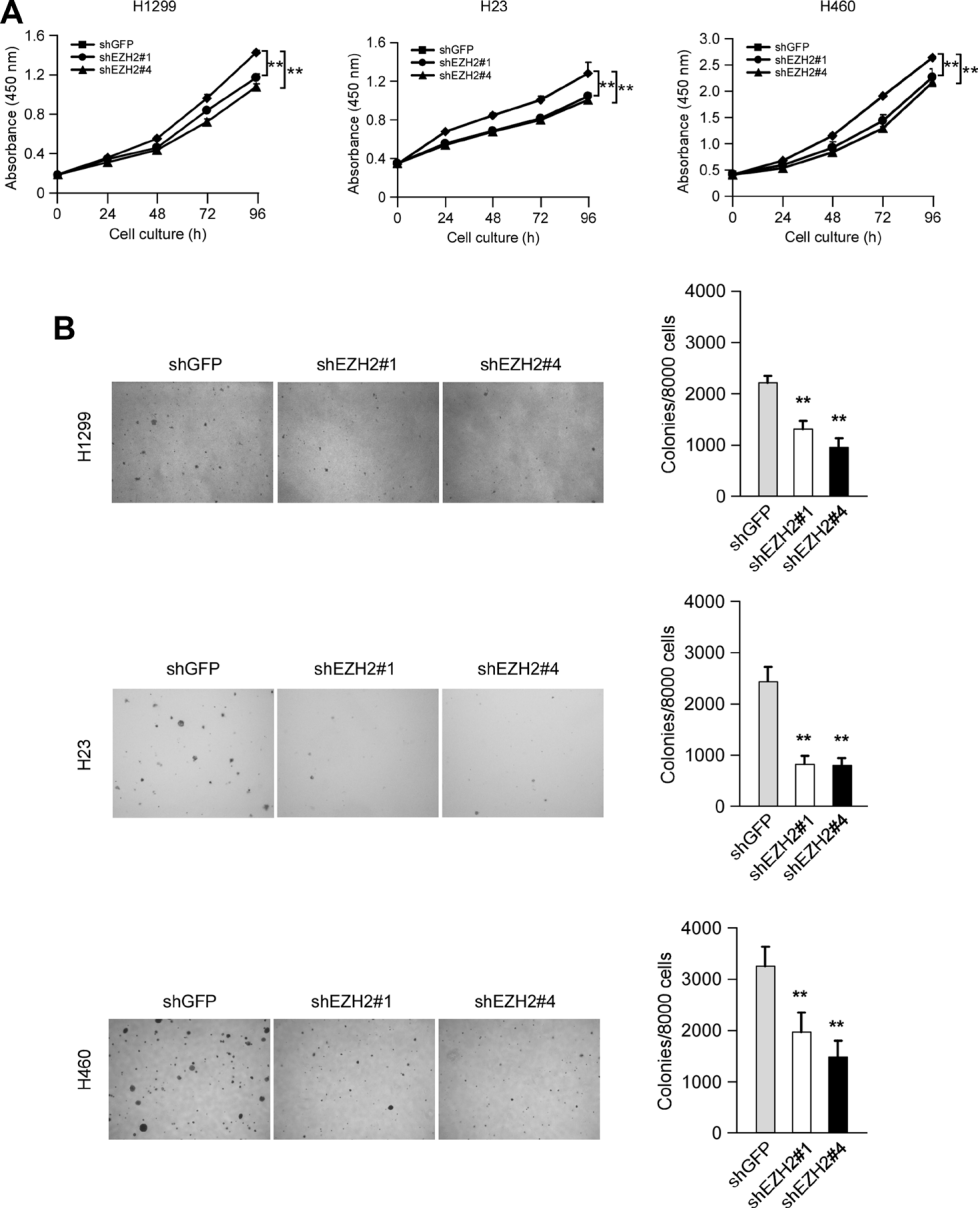
Knocking down the PRC2 catalytic component EZH2 expression reduces human non-small cell lung cancer cells proliferation *in vitro* (**A**) Knockdown of EZH2 attenuates NCI-H1299, NCI-H23 and NCI-H460 anchorage-dependent cell growth. WST-1 assays were performed as described in Materials and Methods. The asterisk (**) indicates a significant (*p* < 0.01) decrease in cell proliferation by knockdown cells. The observed significant difference for H1299, H23 and H460 started from 48 h, 24 h and 48 h, respectively. (**B**) Knockdown of EZH2 attenuates NCI-H1299, NCI-H23 and NCI-H460 anchorage-independent cell growth. Soft agar assays were performed as described in Materials and Methods. The asterisk (**) indicates a significant (*p* < 0.01) decrease in colony formation by knockdown cells.

**Figure 3:**
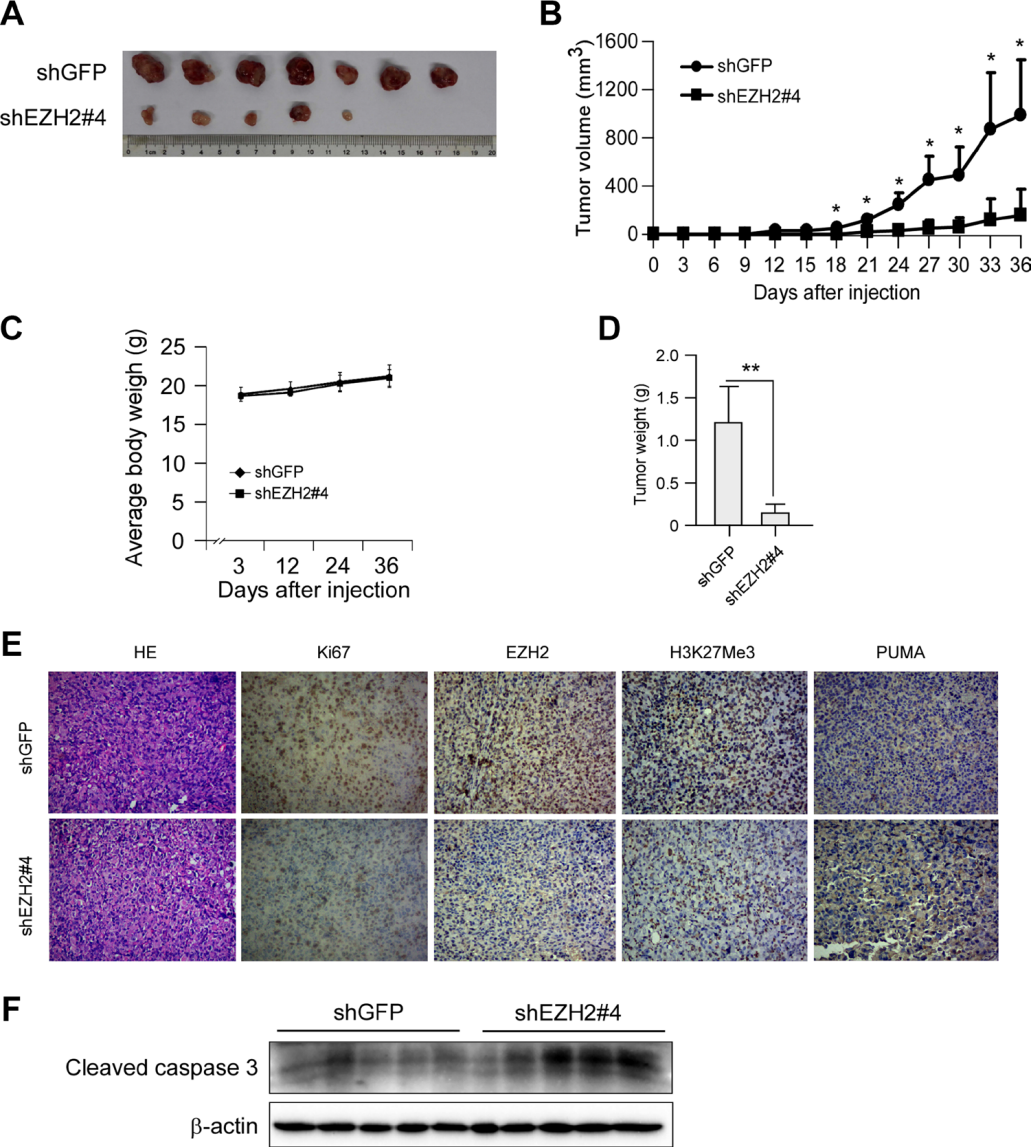
Knocking down the PRC2 catalytic component EZH2 expression inhibits tumor growth *in vivo* (**A**, **B**, **C** and **D**) Knockdown of EZH2 reduces tumorigenic properties of NCI-H1299 NSCLC cells. Photographs of tumors dissected from mice (*n* = 7) injected with H1299-shGFP or H1299-shEZH2#4 cells (A), tumor growth curve (B), average body weight of mice (C) and total average tumor weight (D) from each group were shown. Data are represented as means ± SD of each group. *p < 0.05, **p < 0.01, significant difference compared with the group injected with H1299-shGFP cells. (**E**) Immunohistochemical examination of Ki67, EZH2, histone H3 lysine 27 trimethylation and PUMA in tumor sections from H1299-shGFP or H1299-shEZH2#4 xenograft mice. Representative photographs for each antibody and each group were shown. (**F**) Tumor tissue lysates were prepared and Western blot analysis was done for cleaved caspase 3 to assess the effect of EZH2-knockingdown on cell apoptosis. β-actin was used as a loading control.

To examine whether knockdown of PRC2 expression could influence the growth of NSCLC cells, we generated EZH2, SUZ12 or EED stable knockdown NCI-H1299, NCI-H23 and NCI-H460 cell lines and validated several shRNAs that effectively depleted each PRC2 component after transfection (Supplementary Figure S1). Though knockdown of any single PRC2 component inhibited cell proliferation at varying degrees, results of WST-1 assay indicated that knockdown of EZH2 had a more potent inhibitory effect on cell proliferation than that of SUZ12 or EED (Supplementary Figure S2). Based on this observation as well as EZH2 is generally considered as a crucial component of PRC2 [7], we reasoned that this catalytic PRC2 subunit would most closely reflect the tumorigenic properties of PRC2 and focused mainly on EZH2 in the subsequent studies. To rule out the off-target effect of the shRNA, two sequences, one targeting the *3′UTR* of *Ezh2* gene (shEZH2#1, TRCN0000040073), the other targeting both the *5′UTR* and the coding sequence of *Ezh2* gene (shEZH2#4, TRCN0000040076), were used. The results showed that knockdown of EZH2 in these NSCLC cells suppressed cell proliferation (Figure 2A). Moreover, knocking down EZH2 expression significantly attenuated the colony formation of these NSCLC cell lines in soft agar (Figure 2B). Additionally, we found that knockdown of EZH2 inhibited NCI-H1299 growth in a xenograft mouse model (Figure 3A, 3B, 3C and 3D). Immunohistochemical analysis indicated that knockdown of EZH2 significantly decreased the positive staining of H3K27Me3 and Ki67 (Figure 3E). These results suggest that blocking EZH2 expression significantly reduces the tumorigenic properties of NSCLC cells *in vitro* and *in vivo*. Notably, knockdown of EZH2 resulted in an increase of PUMA staining (Figure 3E). Furthermore, tumor tissue lysates from shGFP-H1299 and shEZH2-H1299 groups were analyzed by immunoblotting for cleaved caspase 3, one of the apoptotic markers [25], to check apoptosis in xenograft tumors. As expected, increased levels of cleaved caspase 3 were observed in shEZH2-H1299 group of tumors compared with shGFP-H1299 control group of tumors (Figure 3F), suggesting EZH2 knocking down caused PUMA induction and inhibited cell proliferation through PUMA-mediated apoptosis.

### Declining EZH2 level accompanies increased PUMA expression in NSCLC cells

**Figure 4:**
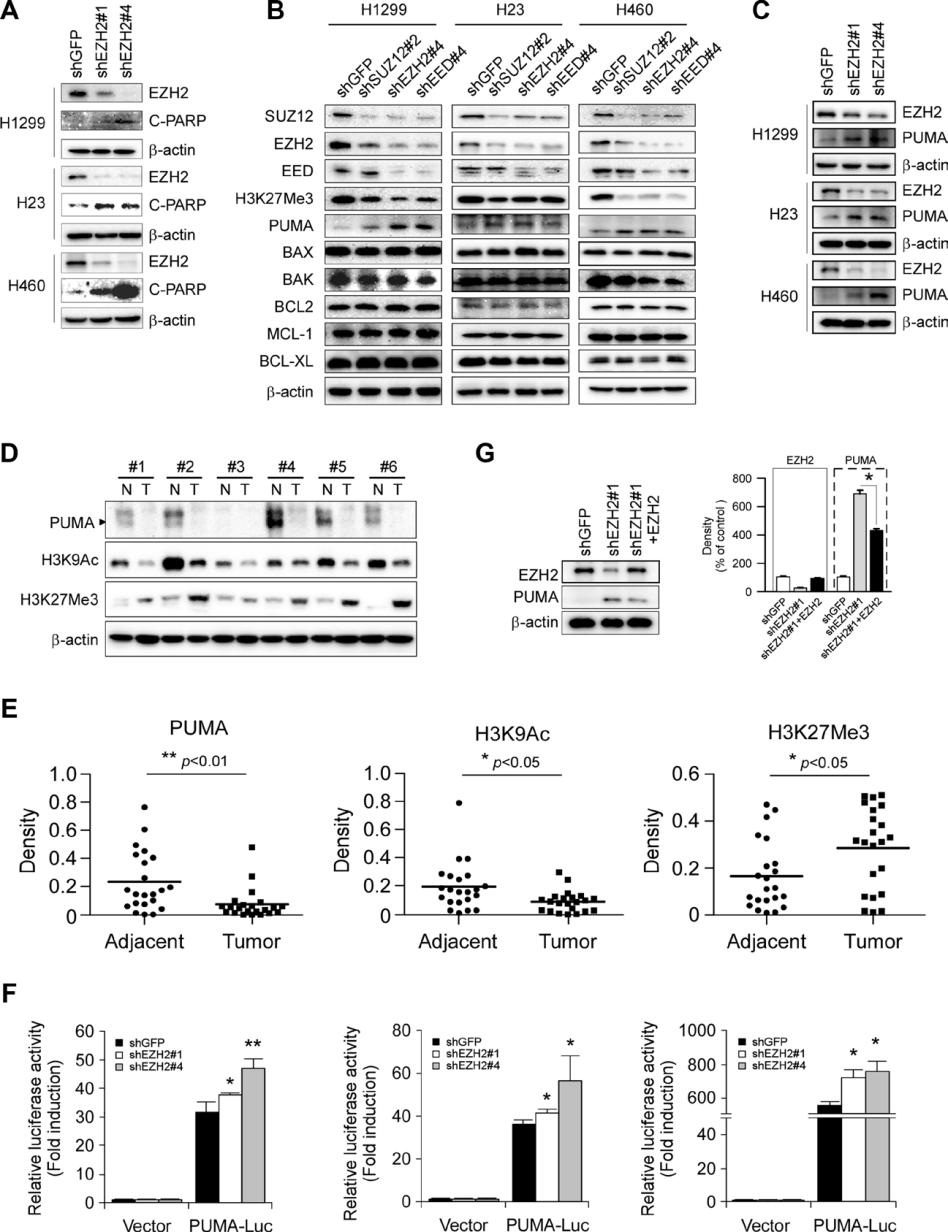
Effects of PRC2 repression on the expression of proapoptotic and antiapoptotic proteins in non-small cell lung cancer cell lines (**A**) Stable knockdown of EZH2 with two shRNA sequences (shEZH2#1 and shEZH2#4) in NCI-H1299, NCI-H23 or NCI-H460 cells and whole cell extracts were analyzed by Western Blotting for the cleavage of PARP. β-actin was used as a loading control. (**B**) Stable knockdown of PRC2 component SUZ12, EZH2 or EED in NCI-H1299, NCI-H23 or NCI-H460 cells and the levels of PRC2 proteins, H3K27Me3, proapoptotic proteins (PUMA, BAX, BAK) and antiapoptotic proteins (BCL2, MCL-1, BCL-XL) were examined by Western blot analysis as indicated. β-actin was used as a loading control. (**C**) Stable knockdown of EZH2 with two shRNA sequences (shEZH2#1 and shEZH2#4) in NCI-H1299, NCI-H23 or NCI-H460 cells and the levels of EZH2 and PUMA were examined by Western blot analysis as indicated. β-actin was used as a loading control. (**D**, **E**) Evaluation the levels of PUMA, H3K9Ac and H3K27Me3 in human NSCLC tissues. PUMA, H3K9Ac and H3K27Me3 levels in six representative NSCLC cases were assessed by Western blot analysis. β-actin was used as a loading control. N, adjacent normal tissues; T, tumor (D). Western blotting determined PUMA, H3K9Ac and H3K27Me3 levels in malignant and the corresponding normal adjacent tissues of 22 NSCLC patients. The intensity was evaluated using Image J (NIH) computer software. *p < 0.05, **p < 0.01, significant difference between groups as indicated (E). (**F**) Dual luciferase reporter assays of plasmid DNA encoding a fragment of human *Puma* promoter in NSCLC cells were performed as described in Materials and Methods. Promoter reporter plasmid was transfected into stable EZH2- knockdown H1299 (left panel), H23 (middle panel) or H460 (right panel) cells. Firefly luciferase readings were normalized to Renilla luciferase to correct for transfection efficiency. *Puma* promoter-driven luciferase activities were expressed as fold induction over the activity of pBV-Luc vector. All experiments were performed in triplicate with at least two independent experiments. *p < 0.05, **p < 0.01, significant difference compared with the shGFP control cells. (**G**) Stable EZH2 knockdown NCI-H460 cells with shEZH2#1 targeting the 3′UTR of *Ezh2* gene were transiently transfected with constructs encoding HA-tagged human EZH2 as described in Materials and Methods. Western blot analysis was done for protein expression levels of EZH2 and PUMA (left panel). β-actin was used as a loading control. The density of each protein was shown (right panel). The intensity was evaluated using Image J (NIH) computer software. *p < 0.05, significant difference between the shEZH2#1 cells and the shEZH2#1 + EZH2 cells.

When present in PRC2, EZH2 trimethylates histone H3 on lysine 27 residue (H3K27Me3), resulting in epigenetic silencing of gene expression. Previous report indicated that decrease of EZH2 expression triggers apoptosis in human epithelial ovarian cancer cells [26]. Indeed, the cleaved PARP, a caspase 3 substrate, was easily detected in H1299-, H23- and H460-shEZH2 cells as compared with their respective shGFP control cells (Figure 4A). These results combined with the observation that knockdown of EZH2 accompanying by an increase of PUMA staining in NCI-H1299 xenograft tumors (Figure 3E) promoted us to investigate the underlying mechanism in more detail. We thus propose a hypothesis that EZH2 knocking down-induced apoptosis (Figure 3F) might contribute to proliferation inhibition and functionally link to an EZH2-mediated epigenetic event. We then sought to determine whether other anti- and proapoptotic members of Bcl-2 family, including BCL2, BCL-XL, MCL-1, BAX and BAK, were the target genes repressed by PRC2/EZH2/H3K27Me3. In NCI-H1299 cells, downregulation of EZH2, SUZ12 or EED induced upregulation of PUMA, without increasing other tested *Bcl-2* family members. In agreement with the previous finding that specific PRC2 HMTase substrate H3K27Me3 [27], a repressive chromatin mark [7], was markedly reduced as a result of PRC2 knockdown (Figure 4B, left panel). Notably, knockdown each of the PRC2 proteins resulted in the downregulation of the other two components (Figure 4B, left panel), results were in line with a report [28] that the protein level of each PRC2 component is dependent on the presence of the other members of the complex. We performed the above experiments and similar results were obtained in NCI-H23 (Figure 4B, middle panel) and NCI-H460 (Figure 4B, right panel) cell lines. Moreover, knockdown of EZH2 with two different sequences both led to PUMA upregulation in NCI-H1299, NCI-H23 and NCI-H460 cell lines (Figure 4C). Additionally, we found that overexpression of EZH2 was accompanied with downregulation of PUMA protein (Figure 4D and 4E). Meanwhile, low levels of H3K9Ac and high levels of H3K27Me3 were observed in the same patient samples where EZH2 is overexpressed (Figure 4D and 4E), which were in consistent with their roles of active and repressive transcriptional marks, respectively. The results were also in agreement with that EZH2 trimethylates H3K27. We further examined the impact of EZH2 on the expression of *Puma* gene in NSCLC cells by luciferase reporter gene assay. Expression of luciferase in the *Puma-Luc* reporter was driven by the *Puma* promoter. Knockdown of EZH2 resulted in increases in luciferase expression of the reporter plasmid in three NSCLC cell lines (Figure 4F), indicating that EZH2 regulated the expression of *Puma* gene in NSCLC cells. Because our shEZH2#1 targets the 3′ untranslated region (3′UTR) and thus is unable to affect the exogenous EZH2, we then carried out rescue experiment with an HA-tagged human EZH2 cDNA. The expression of ectopic EZH2 was confirmed by immunoblotting analysis (Figure 4G). Indeed, the forced expression of EZH2 partially abrogated the increased PUMA upon EZH2 depletion in NCI-H460 cells (Figure 4G). Taken together, the data suggest that the induced expression of proapoptotic PUMA is regulated by EZH2.

### EZH2 directly binds to the promoter region of *Puma* gene and regulates histone H3 modification

**Figure 5:**
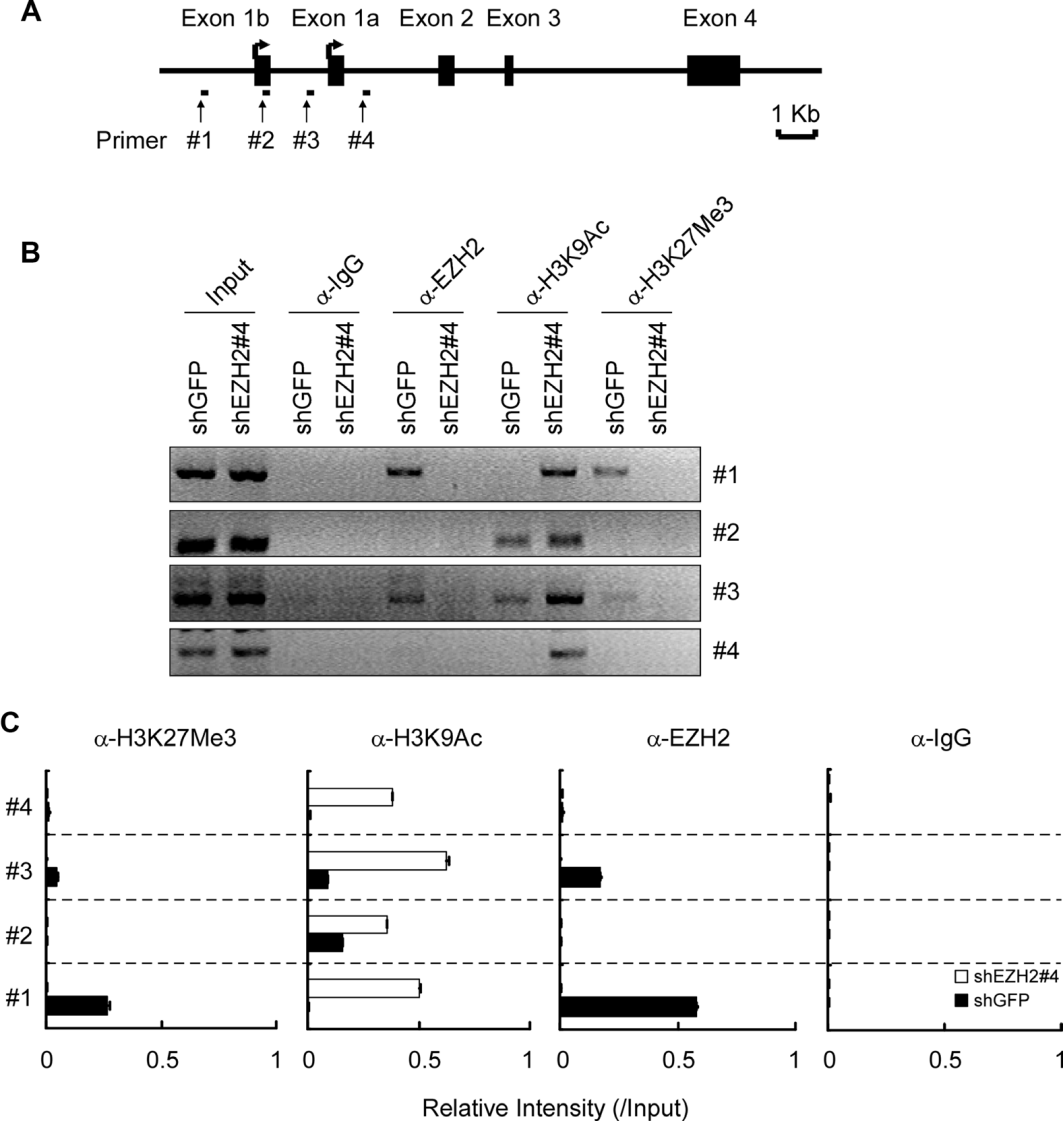
EZH2 binds to the *Puma* gene and regulates histone H3 modification (**A**) Schematic representation of the *Puma* gene locus. The location of primers (#1 to #4) and exons are indicated. (**B**, **C**) H1299-shGFP and H1299-shEZH2 cells were subjected to ChIP assays with an antibody against EZH2, H3K9Ac, H3K27Me3 or normal rabbit IgG. The precipitated DNA fragments were subjected to PCR analysis to test for the presence of sequences corresponding to the *Puma* gene locus. The data were normalized to input and shown as means ± SD of two separate experiments.

To assess the molecular mechanisms underlying the EZH2-involved repression of *Puma* gene, we performed chromatin immunoprecipitation (ChIP) assays with four primer pairs surrounding the core promoter [29] and upstream region of *Puma* gene (Figure 5A). The accumulations of EZH2 were observed around the *Puma* core promoter and the upstream region (Figure 5B and 5C, #1 and #3). Furthermore, to examine whether EZH2 knockdown-induced PUMA upregulation was accompanied by changes in regulatory chromatin mark at the *Puma* locus, ChIP assays with H3K27Me3 and H3K9Ac antibodies were performed. H3K27Me3 at the core promoter and the upstream region of *Puma* gene were observed (Figure 5B and 5C, #1 and #3). As expected, decrease of H3K27Me3 at the *Puma* locus was observed in H1299-shEZH2 cells (Figure 5B and 5C, #1 and #3). By contrast, increase of H3K9Ac, a mark of active transcription at enhancers and promoters [30], was found throughout the *Puma* gene locus tested (Figure 5B and 5C, #1, #2, #3 and #4). Since EZH2 specifically trimethylates H3K27, we conclude that EZH2 loss indirectly affects H3 acetylation at the *Puma* locus. Thus, EZH2 appears to bind to *Puma* gene directly and regulate histone modifications, such as H3K27Me3 and H3K9Ac in NCI-H1299 cells, finally regulates PUMA expression. These results indicate that PUMA is a novel EZH2 target gene, and the upregulation of PUMA might involve in EZH2 knockdown-induced apoptosis in human NSCLC cells.

### Knockdown of EZH2 sensitizes NSCLC cells to cisplatin-induced apoptosis

**Figure 6:**
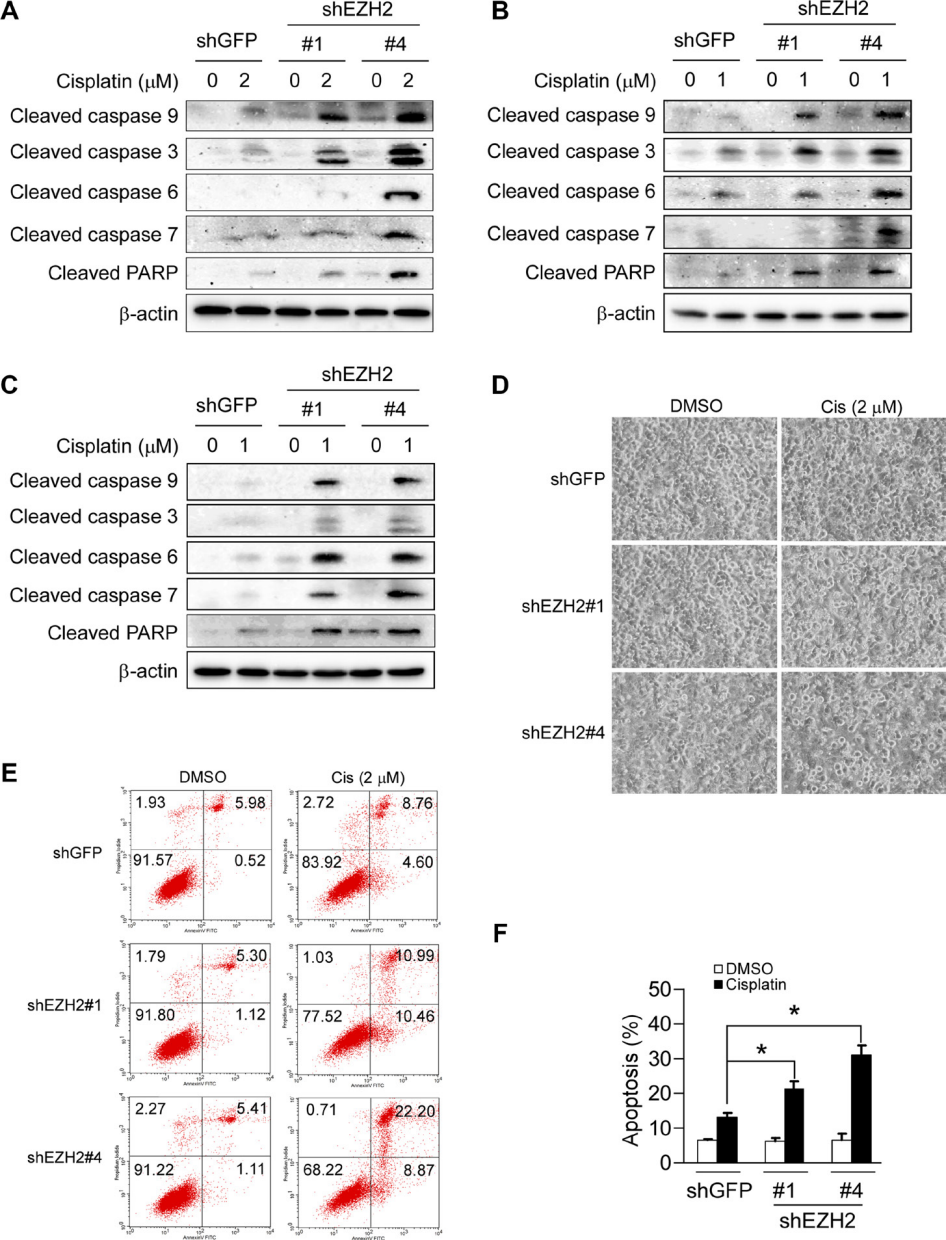
Knockdown of the PRC2 catalytic component EZH2 sensitizes non-small cell lung cancer cells to cisplatin-mediated apoptosis (**A**, **B** and **C**) Stable EZH2 knockdown NCI-H1299 (A), NCI-H23 (B) and NCI-H460 (C) cells were treated without (DMSO) or with the indicated concentrations of cisplatin for 48 h, whole-cell extracts were analyzed by Western blotting. Cleavage of caspase 3, 6, 7, 9 or PARP was detected after cisplatin treatment. β-actin was used as a loading control. (**D**) Stable EZH2 knockdown NCI-H1299 cells were treated without (DMSO) or with 2 μM cisplatin for 48 h, bright field pictures were taken to show cell morphology. (**E**, **F**) Stable EZH2 knockdown NCI-H1299 cells were treated without (DMSO) or with 2 μM cisplatin for 48 h and stained by propidium iodide and Annexin V-FITC, then analyzed by flow cytometry to determine the apoptotic cells. Data represent mean ± SD from two independent experiments. *, *p* < 0.05, significant difference compared with the shGFP control cells.

Studies have shown that PUMA expression could potentiate cisplatin sensitivities in some NSCLC cell lines [31], we thus examined whether EZH2 knockdown-caused PUMA induction could also enhance the efficacy of cisplatin in NSCLC cell lines tested. Immunoblotting results showed that knockdown of EZH2 combined treatment with cisplatin resulted in enhanced cleavage of procaspase 3, 6, 7, 9 or PARP in NCI-H1299 cells (Figure 6A). Similar results were observed in NCI-H23 (Figure 6B) and NCI-H460 (Figure 6C) cells. Furthermore, cell death was assessed by flow cytometry analysis upon Annexin V/PI staining using H1299-shGFP and H1299-shEZH2 cells as model. The results demonstrated that the application of 2 μM cisplatin for 48 h triggered a significant apoptosis when cells were depleted of EZH2 (21.5% for H1299-shEZH2#1, 31.1% for H1299-shEZH2#4 versus 13.4% for H1299-shGFP) (Figure 6D, 6E and 6F). Despite the apoptotic rate of H1299-shEZH2#1 or H1299-shEZH2#4 treated with DMSO was not significantly different from that of H1299-shGFP, there was a slight tendency for increased apoptosis in H1299-shEZH2 cells (Figure 6D and 6F), which was consistent with the data that the cleaved PARP was easily detected in H1299-shEZH2 cells as compared with H1299-shGFP cells (Figure 4A). These results indicate that the increased apoptosis to cisplatin treatment at least partly due to the induction of PUMA following EZH2 depletion in H1299 cells, suggesting that manipulating epigenetic enzymes such as EZH2 can potentiate the anticancer effects of common chemotherapeutic drug cisplatin.

### Augmented EZH2 expression predicts poor overall survival in NSCLC patients

**Figure 7:**
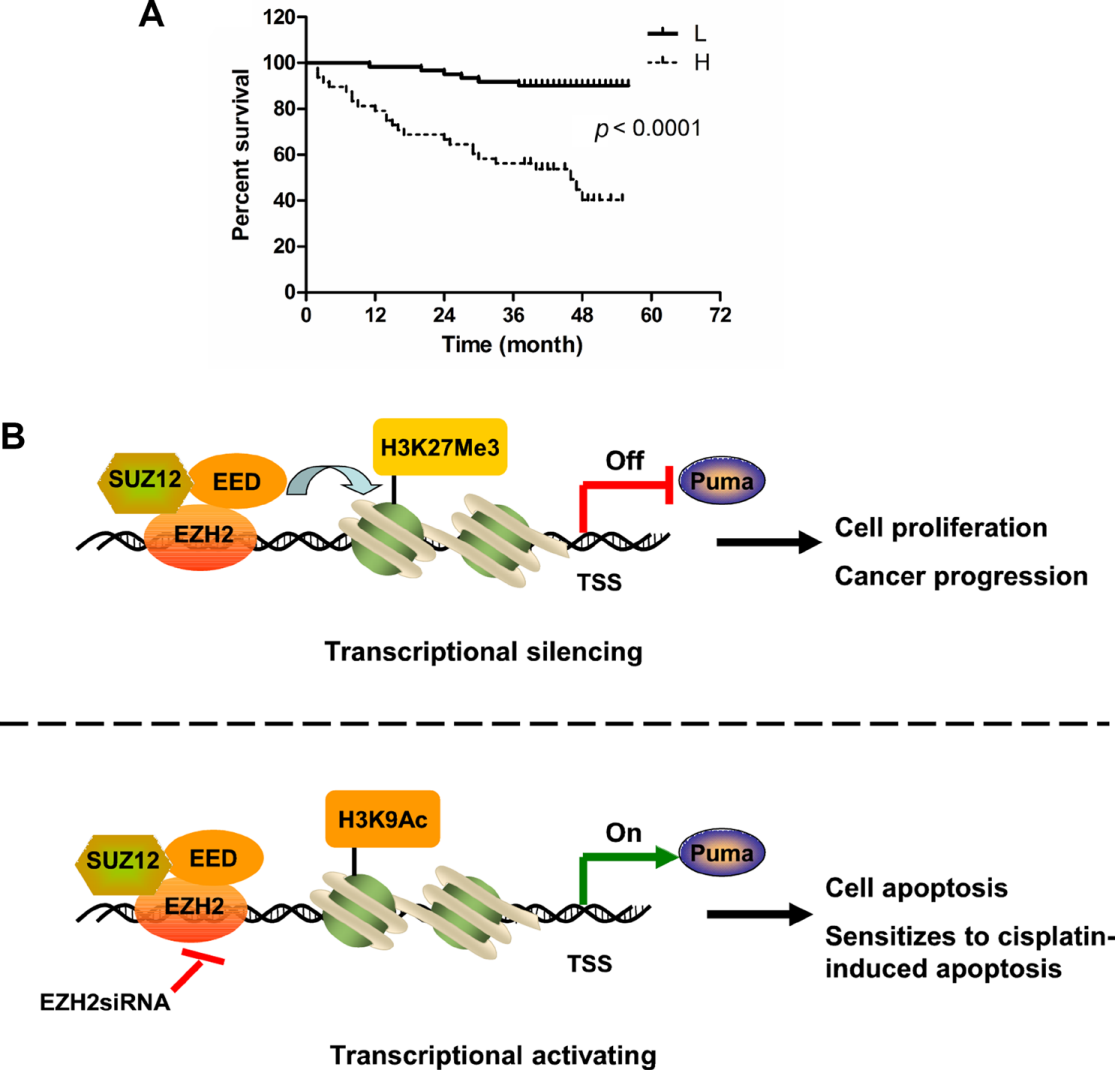
High expression of EZH2 predicts poor overall survival in NSCLC patients (**A**) Overall survival rates of NSCLC patients with high (*n* = 61) or low (*n* =48) expression levels of EZH2 were estimated with the Kaplan-Meier method by log-rank test (*p* < 0.0001). (**B**) Schematic model of *Puma* gene regulation by EZH2. In *Puma* transcriptional silencing cells, EZH2 bound at the *Puma* gene promoter methylates Histone H3 at lysine 27 and prevents *Puma* gene expression. EZH2 overexpression promotes NSCLC progression. Upon EZH2 depletion, H3K27 trimethylation at the *Puma* promoter is reduced whereas H3K9 acetylation at the *Puma* promoter is increased, which induces *Puma* gene expression. Depletion of EZH2 sensitizes NSCLC cells to cisplatin-induced apoptosis through inducing PUMA expression.

Tissue arrays were used to evaluate the expression of EZH2 in NSCLC. Of 109 tumor tissue samples, 61 (55.9%) NSCLC cancers had high EZH2 expression, whereas only 9 (8.3%) had high expression in adjacent non-tumor tissues. These results suggested that EZH2 expression was upregulated in NSCLC compared to non-tumor tissues (*p* < 0.001, Table 1). The association between EZH2 level and clinical features of patients, including age, gender, histopathologic characteristics, lymph node status, initial clinical stage, tumor stage were summarized in Table 2. EZH2 was positively correlated with tumor stage (*p* = 0.027, Table 2). To further explore the relationship between EZH2 expression and patient prognosis, Kaplan-Meier analysis was conducted. Patients were subdivided according to EZH2 IHC scores. Result showed that patients with higher level of EZH2 expression had significantly shorter overall survival (OS) compared with patients whose tumors expressed lower level of EZH2 (*p* < 0.0001, Figure 7A). These findings suggest that elevated EZH2 expression may predict a poor outcome for NSCLC patients.

**Table 1:** Protein expression of EZH2 in NSCLC tissues and adjacent normal tissues

Tissue sample	No. of patients	EZH2		*p*- value
		Low	High	
Tumor	109	48	61	< 0.001*
Adjacent	109	100	9	

Chi-square test.

*p* < 0.05 indicates a significant association among the variables.

**Table 2:** Relationships between the expression of EZH2 and clinicalpathological characteristics in 109 patients with NSCLC

Characteristics	All cases	Expression of EZH2		*p* -value
		Low (*n* = 48)	High (*n* = 61)	
**Gender**				0.656
Male	84	38	46	
Female	25	10	15	
**Age (years)**				0.175
≤ 60	53	20	33	
> 60	56	28	28	
**Pathological grade**				0.379
Well, moderate	81	38	43	
Poor	28	10	18	
**Initial clinical stage**				
≤ IIa	74	29	45	0.153
> IIa	35	19	16	
**Tumor stages**				0.027*
T1 + T2	82	31	51	
T3 + T4	27	17	10	
**Lymph nodes status**				0.236
N0 (negative)	65	25	40	
N1 or above (positive)	44	22	22	

Chi-square test.

*p* < 0.05 indicates a significant association among the variables.

## DISCUSSION

Epigenetic chromatin modification is a major regulator of eukaryotic gene expression [32]. Chromatin modifications, including decreased activating marks and increased repressive marks on histone H3, have been associated with the silencing of genes that regulate important cell functions in cancer. Histone modifications include acetylation, phosphorylation, and methylation, resulting in a combination of histone marks that is known collectively as the histone code [33]. Methylation and acetylation are the two histone modifications that have been clinically associated with pathological epigenetic disruptions in cancer cells. In particular, the loss of methylation and acetylation of specific residues in histone H3 has been identified as a marker of tumor cells [34]. H3K27Me3 is a repressive chromatin mark [7] while H3K9Ac is an epigenetic mark representing transcriptionally active chromatin [35]. We found that EZH2 and associated H3K27Me3 were enriched in the *Puma* locus to suppress PUMA expression (Figure 5B and 5C). EZH2 knockdown caused lack of H3K27Me3 and enrichment of H3K9Ac to the *Puma* promoter (Figure 5B and 5C). As a crucial component of PRC2, EZH2 knockdown might also result in the disruption of this entire repressor protein complex and this expected to effectively remove H3K27Me3 for gene activation. We found that knocking down SUZ12 or EED alone also resulted in PUMA upregulation (Figure 4B). Previous finding showed that the protein level of each PRC2 component is dependent on the presence of the other members of the complex [28]. Whether the effect of SUZ12 or EED on PUMA expression is direct or indirect and whether SUZ12 or EED could bind the *Puma* promoter to regulate histone H3 modification need to be further explored. However, our present study provided the proofs that the expression of PUMA, at least in part, was directly modulated by EZH2 through its bound the *Puma* promoter to regulate the modification of histone H3. We proposed a schematic model of *Puma* gene regulation by EZH2. In *Puma* transcriptional silencing cells, EZH2 bound at the promoter region of *Puma* gene trimethylates H3K27 and prevents *Puma* gene expression. EZH2 overexpression promotes NSCLC progression. Upon EZH2 depletion, H3K27Me3 at the *Puma* promoter is reduced, whereas H3K9Ac at the *Puma* promoter is increased, which induces *Puma* gene expression. EZH2 suppression leads to apoptosis and sensitizes tumor cells to cisplatin-induced apoptosis via upregulation of PUMA expression (Figure 7B). Thus, the occupancy of EZH2 at the *Puma* promoter might be an important mechanism to restrict PUMA induction in cancer cells.

In addition to acting as a transcriptional repressor, EZH2 has been demonstrated to function as a PRC2-independently transcriptional activator. EZH2 physically links β-catenin and TCF on the target gene promoters of cyclin B1 and c-Myc and promotes cell cycle progression in ER-positive MCF-7 cells [7, 36]. Moreover, in ER-negative MDA-MB-231 cells, EZH2 upregulates the expression of NF-κB targets TNF and IL-6 via directly interacting with RelA and RelB, which indicates that EZH2 regulates gene expression in a methyltransferase activity independent manner [7, 37]. In castration-resistant prostate cancer, EZH2 acts as a coactivator for critical transcription factors including the androgen receptor and mediates transcriptional induction [38]. EZH2 functions as a double-facet molecule, either as a transcriptional activator or repressor, depending on its association with other members of the PRC2 complex and cellular context [7]. The dual function transcription regulator with a dynamic activity of EZH2 suggests additional mechanism by which EZH2 promotes NSCLC progression and underscores the need for developing context-specific strategy for therapeutic targeting of EZH2 in NSCLC.

Our results showed that knockdown of EZH2 alone induced a significant inhibition of proliferation (Figure 2 and Figure 3) and only a slight increase of apoptosis (Figure 6D, 6E and 6F), though the cleaved PARP was detected (Figure 4A). Knockdown of EZH2 reportedly triggers apoptosis in human epithelial ovarian cancer cells [26]. This diversity might be due to cells to mount alternative responses to stress in a context dependent manner. Moreover, the result that PUMA upregulation following EZH2 depletion induced only a slight apoptosis (Figure 6D, 6E and 6F), which suggested that the proapoptotic potential of PUMA remains inhibited due to additional control factors. Interestingly, depletion of EZH2 alone to induce PUMA expression greatly enhanced apoptosis triggered by cisplatin at a relatively low concentration in NSCLC cell lines tested (Figure 6). The data suggest that targeting EZH2, which is often overexpressed in cancer, might be a useful approach to sensitize anticancer therapy or overcome drug resistance.

PUMA is one of BH3-only proapoptotic proteins and can bind the multidomain antiapoptotic Bcl-2 family members, including BCL2, BCL-XL and MCL-1 [39]. It functions upstream of BAX and BAK, which are required for commitment to cell death if not suppressed by the binding of antiapoptotic Bcl-2 family members [40–42]. The molecular mechanism through which PUMA regulates apoptosis is still controversial. The current model for PUMA-mediated apoptosis proposed that the interactions of PUMA with antiapoptotic proteins cause displacement of the essential apoptosis effectors BAX and/or BAK, resulting in conformational change, multimerization and mitochondrial translocation of BAX and/or BAK [19]. For example, PUMA has been shown to initiate apoptosis by dissociating BAX from BCL-XL, thereby promoting BAX multimerization and mitochondrial translocation [43]. PUMA is also found to promote BAX translocation to mitochondria both by directly interacting with BAX and by competitive binding to BCL-XL in UV-induced apoptosis [44]. Our results demonstrated that, PUMA upregulation was not accompanied with the upregulations of BCL2, BCL-XL, MCL-1, BAX and BAK upon EZH2 knockdown (Figure 4B), whether BAX or BAK executes its proapoptotic function by conformational change or mitochondrial translocation, and which antiapoptotic Bcl-2 family member interacts with BAK or BAX and thus sequesters BAK or BAX in an inactive protein complex need to be further investigated.

It has been reported that PUMA induction is regulated by not only p53-dependent but also p53-independent mechanisms [20–24]. To determine whether the results obtained from *p*53-null [45] NCI-H1299 cells (Figure 4C, Supplementary Figure S3) can be generalized to *p53* wild-type NSCLC cells, we generated EZH2 knockdown in p53 wild-type [46] NCI-H460 NSCLC cells (Supplementary Figure S3). A tendency for induction of PUMA was observed in NCI-H460 cells (Figure 4C). Similar results were also obtained in p53 mutant [46] NCI-H23 NSCLC cells (Figure 4C, Supplementary Figure S3). Our results indicate that induction of PUMA by EZH2 suppression in these NSCLC cell lines irrespective of their *p53* status. EZH2-based gene therapy might be particularly beneficial to patients whose tumors lost p53 or harbor an inactive p53.

Platinum compounds are the foundation of chemotherapy regimens for NSCLC despite poor response rates and limited response duration [47]. We found that EZH2 knockdown synergized with cisplatin-induced apoptosis and the synergistic apoptosis described above (Figure 6) was corresponding to PUMA induction. In cells stably transfected with EZH2 shRNA thus expressing a higher level of PUMA, the cisplatin treatment resulted in strong cleavage of caspase 3, 6, 7, 9 and PARP (Figure 6A, 6B and 6C), indicating the activation of intrinsic apoptotic process. Inhibitors targeting PRC2 or EZH2 have been developed. DZNep, an S-adenosylhomocysteine (SAH) hydrolase inhibitor, has been characterized as an EZH2 inhibitor that disrupts PRC2 and manifests antitumor activity in a variety of cancers [5, 46, 48]. However, subsequent study demonstrated that DZNep was not selective for EZH2 but rather affected most histone methylation marks that were examined [49]. The inhibitors GSK126 [50, 51], EPZ005687 [52], EPZ6438 [53, 54], El1 [55] and UNC1999 [56] have been independently discovered by multiple groups. All these inhibitors bind to the S-adenosyl methionine (SAM) pocket of the EZH2 SET domain and selectively inhibit EZH2 activity. Among these chemicals the GSK126 and EPZ6438 compounds are currently being evaluated in human clinical trials with advanced solid tumors or with relapsed or refractory B-cell lymphoma [53, 54, 57]. Recently, stabilized a-helix of EZH2 (SAH-EZH2) peptides has been developed, which selectively inhibits H3K27Me3 by disrupting the EZH2-EED interaction and lowering EZH2 expression, a mechanism distinct from that of the above-reported specific EZH2 inhibitors targeting the enzyme catalytic domain [5]. Given the role of EZH2 in lung malignancies and the finding that EZH2 knockdown synergizes with cisplatin-induced apoptosis in NSCLC cells, the combination of small molecule inhibitors of EZH2 with traditional anticancer drugs would be efficacious in treating NSCLC. Such knowledge might help to understand how to use EZH2 inhibitors in combination for cancer treatment.

Our results indicated that the expression of EZH2 significantly correlated with overall survival in NSCLC patients. We found that high levels of EZH2 protein were associated with worse overall survival rate (Figure 7A). In addition to lung cancer, high EZH2 protein expression has been observed in several other malignant tumors, such as gastric cancer, pancreatic cancer and its expression has been reportedly correlated with poor survival in these patients [10–14, 58]. The correlation between high EZH2 expression and poor survival in those studies is in good agreement with our current observations in NSCLC.

To summarize, our study reveals a novel epigenetic regulatory mechanism controlling PUMA expression and the extent of apoptosis observed after EZH2 depletion in response to cisplatin in NSCLC cells. These findings encourage the therapeutic targeting of EZH2 as an important modulator of apoptosis selectively for cancer cells. The synergism between EZH2 inhibition and PUMA induction could be exploited as a plausible strategy to enhance platinum therapeutic efficacy or even to utilize EZH2 inhibitor therapy alone in NSCLCs.

## MATERIALS AND METHODS

### Reagents and plasmid constructs

Cisplatin and chemical reagents, including Tris, NaCl, SDS and DMSO, for molecular biology and buffer preparation were purchased from Sigma-Aldrich (St. Louis, MO). Lentivirus plasmids containing *pLKO.1-shEZH2* (#1, TRCN0000040073; #2, TRCN0000040074; #3, TRCN0000040075; #4, TRCN0000040076; #5, TRCN0000040077), *pLKO.1-shSUZ12* (#1, TRCN0000038724; #2, TRCN0000038725; #3, TRCN0000038726; #4, TRCN0000038727; #5, TRCN0000038728), and *pLKO.1-shEED* (#1, TRCN0000021204; #2, TRCN0000021205; #3, TRCN0000021206; #4, TRCN0000021207; #5, TRCN0000021208) were purchased from Thermo Scientific. The *pCMV-HA* vector (cat#631604) was purchased from CloneTech. The EZH2 expression construct *pCMV-HA-hEZH2* (Addgene plasmid #24230), the luciferase reporter *Puma-Luc* (Addgene plasmid #16591), the *pBV-Luc* construct (Addgene plasmid #16539), *pLKO.1-shGFP* (Addgene plasmid #30323), the lentiviral packaging plasmid *psPAX2* (Addgene plasmid #12260) and the envelope plasmid *pMD2.G* (Addgene plasmid #12259) were available on Addgene (Cambridge, MA). The *Renilla* luciferase reporter construct *pRL-SV40* (Promega) was used as previously described [59].

### Cell lines and cell culture

Cells from American Type Culture Collection (ATCC) were cultured at 37°C in a humidified incubator with 5% CO_2_ according to the ATCC protocols. Cells were cytogenetically tested and authenticated before being frozen. Each vial of frozen cells was thawed and maintained for 2 months (10 passages). Of note, 293T cells were cultured with Dulbecco's Modified Eagle Medium (DMEM) containing 10% FBS and 1% antibiotics. Human NSCLC cells, including NCI-H1299, NCI-H460, NCI-H520, NCI-H23 and NCI-H125, were grown in RPMI-1640 medium supplemented with 10% FBS and 1% antibiotics. A549 human NSCLC cells were cultured with F-12K medium containing 10% FBS and 1% antibiotics. MRC5 human normal lung fibroblasts were cultured with Eagle Minimum Essential Medium supplemented with 10% FBS and 1% antibiotics. The cells were cultured for 36 to 48 hours and proteins extracted for analysis.

### Clinical tissue sample collections

Fresh tumor tissues and the corresponding normal adjacent tissues of the same patient with pathologically and clinically confirmed non-small cell lung cancer by the Department of Clinicopathologic were collected from 22 patients with written informed consent by the Department of Thoracic Surgery, The Second Xiangya Hospital of Central South University, Changsha, Hunan, China. Several small pieces of fresh tumor tissue samples were dissected from the main tumor part of each surgically removed specimen. A portion of tumor and normal adjacent tissues were frozen immediately in liquid nitrogen and then stored at −80°C for protein extraction and analysis of polycomb proteins expression by Western blotting. A portion of tumor and normal adjacent tissues were fixed in formalin solution and sent for histological examination. Prior patient consent and approval from the Hospital's Research Ethics Committee were obtained for the use of these clinical materials for research purposes. All the patients received no treatment before surgery.

### Lentiviral infection and transient transfection

The generation of gene stable knockingdown cell lines was performed as described previously [60, 61]. Briefly, to generate EZH2, SUZ12 and EED knockingdown cells, *pLKO*.*1-shGFP*, *pLKO*.*1-shEZH2*, *pLKO*.*1-shSUZ12* or *pLKO*.*1-shEED* lentivirus plasmids were cotransfected into 293T cells with *psPAX2* and *pMD2.G*. Viral supernatant fractions were collected at 48 hours after transfection and filtered through a 0.45 μm filter followed by infection into NCI-H1299, NCI-H460 or NCI-H23 cells together with 6 μg/mL polybrene. At 16 hours after infection, the medium was replaced with fresh medium containing 2 μg/mL puromycin and cells were incubated for another 3 days. For transient expression of EZH2 by plasmid transfection, H460-shGFP and H460-shEZH2#1 cells in 6-well plates were transiently transfected 4.0 μg *pCMV-HA* or *pCMV-HA-hEZH2* with Lipofectamin 2000 (cat#11668-019, Invitrogen, Carlsbad, CA) for 48 h following the manufacturer's instructions. The ectopic expression of EZH2 was verified by Western blot analysis. For transient expression of the human *Puma* promoter, NSCLC cells growing on 24-well plates were co-transfected with firefly luciferase reporter plasmids and the *Renilla* luciferase reporter construct, lysates were collected 48 h after transfection with Lipofectamine 2000 (cat#11668-019, Invitrogen, Carlsbad, CA). Firefly luciferase and *Renilla* luciferase activity was determined using the Dual-Luciferase reporter assay system (Promega) with a GloMax 20/20 luminometer (Promega). Firefly luciferase readings were normalized to Renilla luciferase to correct for transfection efficiency. The data are represented as the fold induction compared to the *pBV-Luc* vector. All experiments were performed in triplicate with at least two independent experiments.

### Protein preparation and Western blotting

Frozen tissue samples were sectioned into small pieces and dissolved in lysis buffer containing 50 mM Tris-Cl (pH 8.0), 150 mM NaCl, 0.1% SDS, 100 μg/ml phenylmethylsulfonyl fluoride, 2 μg/ml aprotinin, 2 μg/ml leupeptin and 1% NP-40. The samples were homogenized, sonicated and kept on ice for 30 minutes. After centrifugation, the supernatant was collected for immunoblotting analysis. Cultured cells were harvested and whole cell lysates were prepared according to the method previously described [60]. Protein concentration was determined using the BCA Assay Reagent (cat#23228, Pierce, Rockford, IL). Western blotting was performed as previously described [60]. Primary antibodies were used for immunoblotting: EZH2 (#5246), SUZ12 (#3737), Tri-Methyl-Histone H3 (Lys27) (#9733), Acetyl-Histone H3 (Lys9) (#9649), Histone H3(#4499), MCL-1 (#5453), BCL2 (#2870), BCL-XL (#2764), PUMA (#4976), BAX (#5023), BAK (#6947), cleaved caspase-3 (#9664), cleaved caspase-6 (#9761), cleaved caspase-7 (#8438), cleaved caspase-9 (#9505), and cleaved PARP (#5625) from Cell Signaling Technology; EED (#17-663) from Merck Millipore; β-actin (A5316) from Sigma-Aldrich. Secondary antibodies were anti-rabbit IgG HRP (#7074) and anti-mouse IgG HRP (#7076) and purchased from Cell Signaling Technology. Antibody conjugates were visualized by chemiluminescence (ECL; cat#34076, Thermo).

### Immunohistochemical (IHC) staining

Tumor tissues obtained from NSCLC patients or euthanized xeno-grafted mice were embedded in paraffin and subjected to immunohistochemistry staining with specific antibodies against EZH2 (1:100, #5246, Cell Signaling Technology), SUZ12 (1:50, ab12073, Abcam), EED (1:50, ab96801, Abcam), PUMA (1:100, ab33906, Abcam), Tri-Methyl-Histone H3 (Lys27) (1:100, #9733, Cell Signaling Technology) or Ki67 (1:200, ab16667, Abcam) according to the DAKO system protocol. Hematoxylin was used for counterstaining. Slides were viewed and photographed under a light microscope, and analyzed using Image-Pro Plus Software (version 6.2) program (Media Cybernetics). Human NSCLC tissue arrays (HLug-Ade150Sur-02 and HLug-Squ150Sur-02) were purchased from Shanghai Outdo Biotech Co.,Itd. (Shanghai, China). The arrays included 49 cases of adenocarcinoma and 60 cases of squamous cell carcinoma with clinical stages and follow-up records for 5 year. The latest follow-up information was updated in July 2012, overall survival (OS) was defined as the time from completion of therapy to the date of death or when censored at the latest date if patients were still alive. EZH2 expression was scored according to staining intensity and the percentage of positive cells as previously described [62]. The percentage of positive cells was scored as follows: 0, no positive cells; 1, ≤ 10% positive cells; 2, 10–50% positive cells; 3, > 50% positive cells. Staining intensity was scored as follows: 0, no staining; 1, faint staining; 2, moderate staining; 3, dark staining. Comprehensive score = staining percentage × intensity. EZH2 expression: < 2 low expression, ≥ 2 high expression.

### Cell growth assays

Cells were seeded at a density of 2 × 10^3^ cells per well in 96-well plates in 100 μl of RPMI 1640 medium containing 10% FBS and incubated in a 37°C, 5% CO_2_ incubator. After culturing for another 24, 48, 72 or 96 h, 10 μl of the WST-1 Reagent (Roche, Mannheim, Germany) were added to each well and cells were incubated for 2 h at 37°C. The absorbance of the cellular reduction of WST-1 to formazan was measured at 450 nm as previously described [60]. Three independent experiments were performed in triplicate.

### Anchorage-independent cell growth assay

Cells (8 × 10^3^ per well) were seeded into 6-well plates with 0.3% Basal Medium Eagle agar containing 10% FBS and cultured. The cultures were maintained at 37°C in a 5% CO_2_ incubator for 2 or 3 weeks and colonies were counted under a microscope as previously described [60].

### Apoptosis assays

NSCLC cells were seeded into 6-well plates in RPMI 1640 containing 10% FBS. After culturing for 12 h, different concentrations of cisplatin were added to each well and left on the cells for 2 days. After treatment, attached and floating cells were harvested. For apoptosis analysis, the cells were suspended in 1 × 10^6^ cells/ml, and 5 μl Annexin V and Propidium Iodide staining solution were added to 300 μl of the cell suspension. After incubated 10–15 minutes at room temperature in the dark, stained cells were assayed and quantified using a FACSort Flow Cytometer (BD, San Jose, CA, USA). Each experiment was done in triplicate and repeated at least twice.

### Chromatin-immunoprecipitation (ChIP) assays and polymerase chain reactions (PCR)

ChIP assays were performed as previously described [60] . Briefly, H1299-shGFP and H1299-shEZH2#4 cells were cross-linked with 1% formaldehyde, neutralized with 125 mM glycine, harvested, and disrupted by sonication to fragments with an average size of ~500 bp. The chromatin of H1299-shGFP or H1299-shEZH2#4 cells was pre-cleared with 30 μl protein G agarose/salmon sperm DNA (#16-201, Upstate) and incubated with 2 μg of EZH2 (#5246, Cell Signaling Technology), Tri-Methyl-Histone H3 (Lys27) (#9733, Cell Signaling Technology), Acetyl-Histone H3 (Lys9) (#9649, Cell Signaling Technology) or normal rabbit IgG (#NI01, Calbiochem) antibody at 4°C overnight. The immunocomplexes were pulled down with 30 μl dynabeads Protein G (#100.03D, Invitrogen). The beads were collected on a magnetic device and washed with ChIP wash buffer and TE buffer (10 mM Tris-HCl, pH 8.0, 1 mM EDTA). Cross-links for both ChIP and input DNA were reversed at 65°C for 5 h and DNA was purified with E.Z.N.A Cycle-pure Kit (Omega BIO-TEK, Norcross, GA, USA). In order to determine the optimal PCR cycle numbers, a constant amount of each ChIP-DNA sample was used in PCR reactions and the cycle numbers were varied between 25 and 40 (25, 28, 32, 36, 38, 40) in the preliminary experiments. Each PCR product of 36 cycles showed a good detectable signal and was in the linear range. Thus, PCR thermocycling conditions for all ChIP-DNA samples are 95°C for 5 min followed by 36 cycles of 95°C for 30 sec, 58°C for 30 sec, 72°C for 30 sec and an extension for 10 min at 72°C. Equal amount of each ChIP-DNA was used as a template for PCR. PCR products were analyzed by electrophoresis on a 2% agarose gel and visualized by ethidium bromide staining. The primer pairs (Supplementary Table S1) were used to amplify the *Puma* core promoter and the upstream regions present in the immunoprecipitated DNA. The data are expressed as enrichment related to input [60].

### *In vivo* tumor growth assay

All mice were maintained and manipulated according to strict guidelines established by the Medical Research Animal Ethics Committee, Central South University, China. Xenograft tumors were established by s.c. injection of H1299-shGFP or H1299-shEZH2#4 NSCLC cells (3 × 10^6^) into the flank of 6-week-old athymic nude mice (*n* = 7). Mice were weighed and tumors measured by caliper every three day. Tumor volume was calculated from measurements of 2 diameters of the individual tumor according to the following formula: tumor volume (mm^3^) = (length × width × width/2) [63]. Mice were monitored until day 36 and at that time mice were euthanized and tumors extracted.

### Statistical analysis

Statistical analysis was performed with SPSS 16.0 (SPSS, Inc, Chicago, IL). Survival curve was estimated using the Kaplan-Meier method. The log-rank test was used to identify statistically significant differences between survival curves. The association between clinicopathologic factors and EZH2 level was evaluated using Chi-square test. The data from *in vitro* study were expressed as means ± SD as indicated. All *p* values quoted were two sided and *p* < 0.05 was considered statistically significant.

## SUPPLEMENTARY MATERIALS FIGURES AND TABLE


