# Supplementary material for: EZH2-mediated Puma gene repression regulates non-small cell lung cancer cell proliferation and cisplatin-induced apoptosis
Source: Oncotarget. 2016 Jul 26;7(35):56338–54. doi: 10.18632/oncotarget.10841 (PMC5302918; doi:10.18632/oncotarget.10841)
Supplement: Supplementary file 1 [file oncotarget-07-56338-s001.pdf]

EZH2-mediated *Puma* gene repression regulates non-small cell lung cancer cell proliferation and cisplatin-induced apoptosis

Supplementary Materials

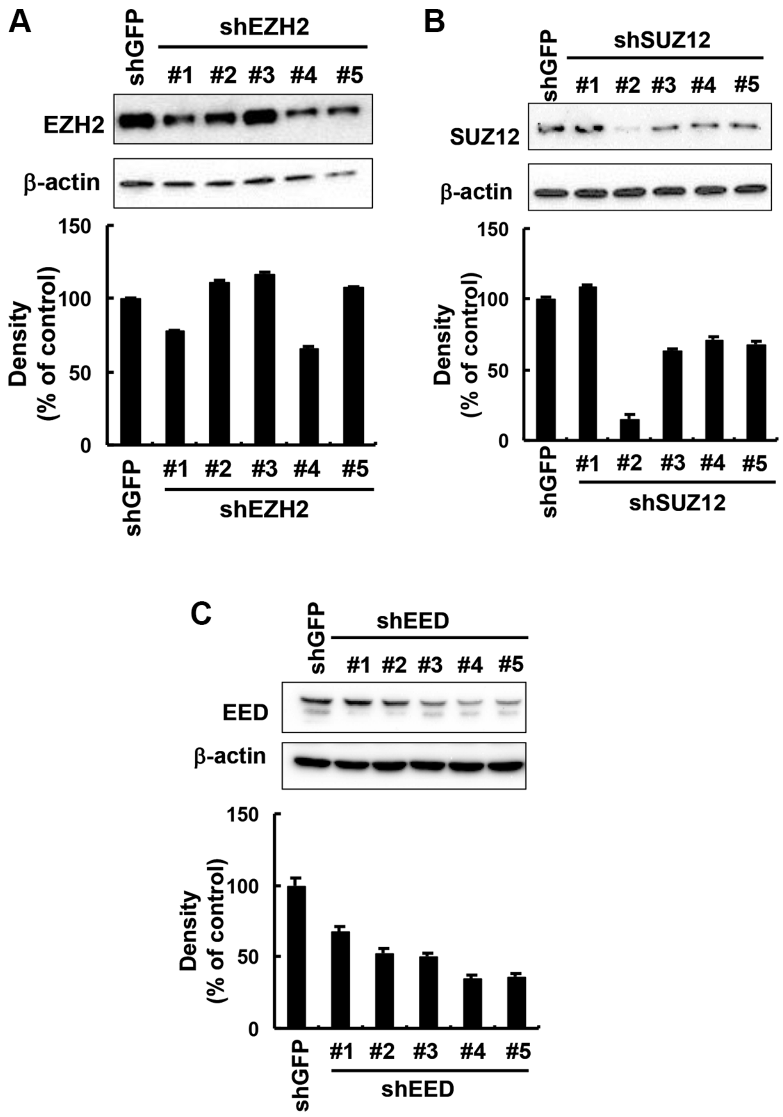

Supplementary Figure S1: Validation of the PRC2 component knockdown by their shRNAs in human non-small cell lung cancer cells. (**A**, **B** and **C**) NCI-H1299 cells were transduced with lentiviral short hairpin RNAs (shRNAs) targeting GFP control (shGFP), SUZ12 (#1 to #5), EZH2 (#1 to #5) or EED (#1 to #5). The knockdown of SUZ12, EZH2 or EED expression is confirmed by Western blot analysis 3 d after shRNA transduction. ß-actin was used as a loading control.

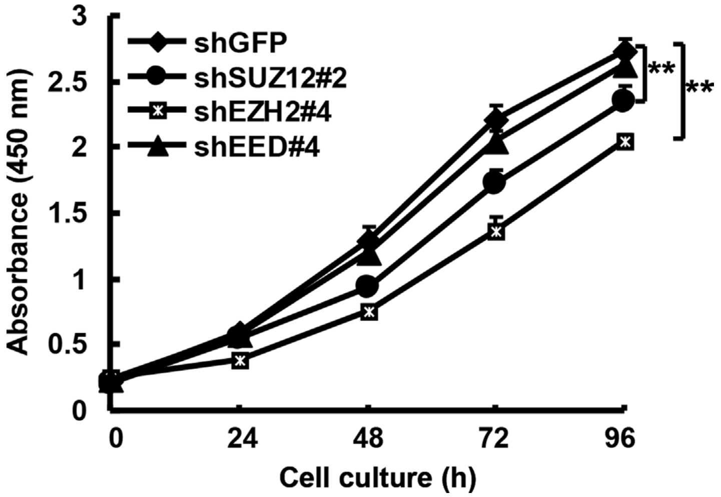

Supplementary Figure S2: Effect of the PRC2 component knockdown individually on non-small cell lung cancer cell proliferation. Knockdown of SUZ12, EZH2, or EED attenuates NCI-H1299 anchorage-dependent cell growth. WST-1 assays were performed as described in Materials and Methods. The asterisk (**) indicates a significant ($p < 0.01$) decrease in cell proliferation by knockdown cells.

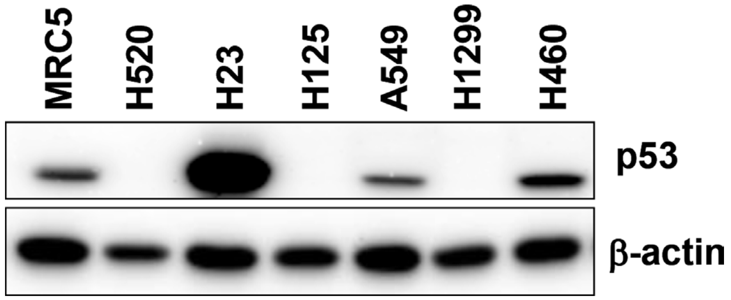

Supplementary Figure S3: Different p53 status in human non-small cell lung cancer cell lines. Western blot analysis was performed to examine p53 expression with an anti-p53 antibody (sc-126, Santa Cruz Biotechnology) in several NSCLC cell lines and normal MRC5 lung cells. ß-actin was used as a loading control.

Supplementary Table S1: Primers for amplification of the *Puma* promoter by PCR

Primer	Sense sequence (5′to3′)	Antisense sequence (5′to3′)	Size (bp)
#1	GCAGGAGTTTGAAACCAGCC	TCACATCCCAACCAACTTGC	217
#2	GGTCCCCTGCCAGATTTGT	AGAAACTGACCACCCACACA	124
#3	GCGAGACTGTGGCCTTGTGT	GACAGTCGGACACACACACT	151
#4	GCGCACAGGTGCCTCGGC	TGGGTGTGGCCGCCCCT	174
